# Methotrexate and Valproic Acid Affect Early Neurogenesis of Human Amniotic Fluid Stem Cells from Myelomeningocele

**DOI:** 10.1155/2017/6101609

**Published:** 2017-09-13

**Authors:** Vardine Sahakyan, Enrico Pozzo, Robin Duelen, Jan Deprest, Maurilio Sampaolesi

**Affiliations:** ^1^Department of Development and Regeneration, Stem Cell Institute, Stem Cell Biology and Embryology Unit, Translational Cardiomyology Laboratory, KU Leuven, Leuven, Belgium; ^2^Department of Development and Regeneration, Division Woman and Child, University Hospitals Leuven, KU Leuven, Leuven, Belgium; ^3^Institute for Women's Health, University College London, London, UK; ^4^Department of Public Health, Experimental and Forensic Medicine, Division of Human Anatomy, University of Pavia, Pavia, Italy

## Abstract

Myelomeningocele (MMC) is a severe type of neural tube defect (NTD), in which the backbone and spinal canal do not close completely during early embryonic development. This condition results in serious morbidity and increased mortality after birth. Folic acid significantly reduces, and conversely, folate antagonist methotrexate (MTX) and valproic acid (VPA) increase the occurrence of NTDs, including MMC. How these pharmacological agents exactly influence the early neurulation process is still largely unclear. Here, we characterized human amniotic fluid-derived stem cells (AFSCs) from prenatally diagnosed MMC and observed an effect of MTX and VPA administration on the early neural differentiation process. We found that MMC-derived AFSCs highly expressed early neural and radial glial genes that were negatively affected by MTX and VPA exposure. In conclusion, we setup a human cell model of MMC to study early neurogenesis and for drug screening purposes. We also proposed the detection of early neural gene expression in AFSCs as an additional MMC diagnostic tool.

## 1. Introduction

Myelomeningocele (MMC) is the most common form of neural tube defects (NTDs) with an average worldwide incidence of 4 per 10,000 live births [1]. It is caused by defective fusion of neural folds during day 25–28 of gestation, leading to the protrusion of dysplastic meninges and spinal cord from the spinal canal in a cyst-like sac (reviewed in [1]). During pregnancy, fetuses with MMC develop progressive motor and sensory deficits and hindbrain herniation (Arnold-Chiari Malformation II) and in some variable degrees of ventriculomegaly. Depending on the level of the lesion, there will be bowel and bladder dysfunctions. Some children may have an intellectual deficit, in particular in case of complicated hydrocephaly [1, 2].

The etiology of MMC is unclear, with both environmental factors and genetic variations predisposing to the condition [3, 4]. The use of folic acid (FA) supplementation during pregnancy reduces the risk of NTDs up to 70% [5, 6], while prenatal exposure to folate antagonists, including methotrexate (MTX) and valproic acid (VPA), increases the risk of NTDs [7, 8]. Hence, daily FA intake of at least 400 micrograms is recommended to all women of reproductive age to prevent NTD from occurring [4, 5].

Folate coenzymes play an important role in several crucial processes, including nucleotide biosynthesis, generation of methyl donors and cell proliferation [9–11]. Both MTX and VPA are known to influence enzymes crucial for the folate metabolic pathway [12, 13]. Yet, the exact mechanisms through which MTX and VPA cause NTDs remain unclear [5].

The embryonic neural plate and neural tube are formed from neuroepithelial (NE) cells. These polarized cells actively proliferate and express the earliest marker for neural plate SOX2 and an intermediate filament protein NESTIN. Subsequently, immature neurons expressing neural *β*III-tubulin and radial glial (RG) cells expressing BLBP appear for further neural tube organization [14–18]. FA stimulates the proliferation and differentiation of neural stem cells (NSCs), whereas MTX and VPA impair cell proliferation of embryonic NSCs and amniotic fluid-derived neural stem cells, respectively [19, 20]. It is likely that undesirable effects of VPA and MTX on the developing neural tube are due to the influence of genes crucial for neural tube development, including Ki67, SOX2, and NESTIN [7, 20]. Obviously, the assessment of undesirable effects of drugs in pregnancy is limited for ethical reasons, with limited options to enroll pregnant women in clinical trials [20]. Thus, retrospective epidemiological studies or experimental animal models remain the main sources for studying drug effects. Therefore, novel platforms, such as human MMC cell models, are being developed to study the disease characteristics and early neurulation process and to reveal the effects of novel therapeutic drugs.

The amniotic fluid has been shown to contain a heterogeneous population of cells [21, 22] that may be used as an invaluable source of patient-derived cells. In recent years, there has been growing interest in amniotic fluid-derived stem cells (AFSCs) due to their possible applications as clinical therapeutic tools for disease modeling, drug screening, and regenerative medicine. AFSCs are a subset of multipotent fetal stem cells that retain the ability to differentiate various cell types [21–24]. Previous studies have demonstrated successful differentiation of AFSCs to neural-like cells [25–28], and pharmacological agents, including VPA, have been shown to affect their characteristics [20].

In this study, we isolated and characterized AFSCs from fetuses prenatally diagnosed with MMC. Following induction into early neural differentiation, we described the effects of MTX and VPA on MMC-derived AFSCs compared to healthy AFSCs. This study offers a novel cell-based model to investigate human MMC characteristics and for diagnostics and drug screening purposes.

## 2. Material and Methods

### 2.1. Amniotic Fluid Samples Collection

The Ethics Committee of the University Hospital Leuven and KU Leuven approved the study (ML9167). Amniotic fluid samples were collected from 7 patients presenting with a fetus with MMC at 23–25 gestational weeks (term = 40 weeks). Additionally, amniotic fluid samples were obtained at 19–21 gestational weeks from three patients with healthy fetuses.

### 2.2. Isolation and Culture of AFSCs

Isolation of the AFSCs from amniotic fluid samples was performed by using previously published protocols [29] with few adjustments. Briefly, amniotic fluid was centrifuged for 10 minutes at 800 rpm; the cell pellet was resuspended in mesenchymal basal medium, containing DMEM (Invitrogen), 20% Fetal Bovine Serum (FBS, Invitrogen), 1% Pen/Strep (Thermo Fisher Scientific), and 2 mM L-glutamine (Thermo Fisher Scientific). Cells were incubated at 37°C with 5% humidified CO_2_. After 36 hours, cells were washed with Phosphate-Buffered Saline (PBS, Invitrogen) to remove nonadherent cells, and the culture medium was replaced by fibroblast growth medium, containing KO-DMEM (Invitrogen), 2 mM L-glutamine, 10% FBS and 1% Pen/Strep. The medium was refreshed every three days. Cells were daily monitored by light microscopy. When reaching 80–90% of confluence, cells were detached by enzymatic treatment, using TrypLE (Gibco) and expended.

### 2.3. Cell Viability Assays

Viability assays were performed on AFSCs after each passage, exposed or not to VPA and MTX. Briefly, 10 *μ*L of the cell pellet from each MMC and healthy line was resuspended in 10 *μ*L of Trypan Blue (Life Technologies) and inserted in an automatic cell counter machine (Invitrogen). The number of live/dead cells and viability were evaluated.

### 2.4. Flow Cytometry Analysis

Amniotic fluid cells were harvested from culture and dissociated by EDTA Accutase (Thermo Fisher Scientific) for 3 minutes at 37°C. Samples were collected in staining buffer (Hanks' Balanced Salt Solution, HBSS; Thermo Fisher Scientific) with CaCl2 and MgCl2 supplemented with 2% FBS, 10 mM HEPES, and 10 mM NaN3 (both from Sigma-Aldrich; pH 7.2). Cells were quantified and 100,000 cells from each line were stained for 30 minutes at room temperature (RT) with the following antibodies: CD44 (0.25 *μ*g), CD117 (0.25 *μ*g), CD105 (1 *μ*g), CD73 (0.125 *μ*g), and CD90 (1 *μ*g/mL, all from Thermo Fisher Scientific, Bioscience). After incubation, cells were washed with PBS, centrifuged for 5 minutes at 800 rpm, and the cell pellet was resuspended in 200 *μ*L staining buffer. Cells were analyzed and quantified by flow cytometry (BD FACSCanto I or II with HTS; BD Biosciences) and FlowJo Software (FlowJo LLC).

### 2.5. Quantitative Real-Time PCR

The GeneElute Mammalian Total RNA Miniprep Kit (Sigma-Aldrich) was used for RNA extraction, following the manufacturer's protocol. Reverse transcription was performed using 1 *μ*g of RNA by SuperScriptTM III First-Strand Synthesis SuperMix Kit (Invitrogen) and diluted in DEPC water. The oligonucleotide primer sequences are listed in Supporting Information Table S1 available online at https://doi.org/10.1155/2017/6101609 (all from IDT supplier). SYBR Green (Invitrogen) was used to perform the RT-qPCR gene expression on real-time system Realplex2 Master Cycler (Eppendorf) or on the ViiA7 Real-Time PCR system (Invitrogen).

### 2.6. Optimization of MTX and VPA Concentration for Cell Culture Use

Concentrations of both pharmacological agents were setup considering previous studies [20] and dose-response experiments (Supporting Information Figures 1(a) and 1(b)). Increasing concentrations were tested for each pharmacological agent, and cell viability assay analyses were performed. 0.25 *μ*M MTX and 1 mM VPA did not affect live/dead viability assays and morphology of the cells. Thus, these concentrations were chosen to perform experiments. Pharmacological agents were added only during the first three days of neural induction.

### 2.7. Neural Induction from MMC-AFSCs

For the enhancement of neural induction properties, MMC-AFSCs and healthy AFSCs were detached by enzymatic treatment, using Accutase (Thermo Fisher Scientific). Cells were plated on laminin-coated plates (Sigma-Aldrich) in a mesenchymal basal medium as described above. The next day, medium was changed to neuronal induction medium, containing DMEM-F12 (Gibco), Neurobasal Medium (Gibco), N-2 Supplement (Gibco), B-27 Supplement (Gibco), human insulin solution (Sigma-Aldrich), L-glutamine, 0.1 mM NEAA (Thermo Fisher Scientific), 2-mercaptoethanol (Thermo Fisher Scientific), basic fibroblast growth factor (bFGF, 20 ng/ml, Lonza), as well as the epithelial growth factor (EGF, 20 ng/ml, Lonza). The medium was refreshed every other day. Cells were cultured for 15 days before processing for immunofluorescence analysis.

### 2.8. Immunofluorescence Staining

AFSCs were fixed with 4% PFA for 10 minutes at RT. Permeabilization was performed in Triton-X-100 at RT for 30 minutes. Cells were washed twice and blocked in 10% donkey serum (Sigma-Aldrich) for 30 minutes. Incubation with the following primary antibodies SOX2 (1 : 200, Santa Cruz, sc-8628), NESTIN (1 : 200, Covance, MMS-570P), *β*III-tubulin (1 : 1000, Santa Cruz, sc-21,705), BLBP (1 : 2000, Chemicon, AB9558), PAX3 (1 : 1000, R&D Systems, MAB2457), and Ki67 (1 : 50, Dako, M7240) was performed overnight at 4°C followed by the secondary antibodies (1 : 500, Invitrogen) for 30 minute at RT. Nuclei were counterstained with Hoechst (33,342, Thermo Fisher Scientific; 1 : 3000) for 2-3 minutes, and coverslips were mounted on slides with FluorSave (Merck). Fluorescent imaging was obtained by using Eclipse Ti Microscope (Nikon). Image-Pro Plus 6.0 software was used for enumeration of SOX2, NESTIN, *β*III-tubulin, BLBP, PAX3, and Ki67 positive cells from MMC-AFSCs and healthy AFSCs, exposed or not to MTX and VPA.

### 2.9. Statistical Analysis

Data were analyzed using GraphPad Prism 6. All data were obtained from 4 independent experiments and reported as mean ± standard deviation (SD). In particular, 7 MMC-AFSC lines and 3 healthy AFSC lines have been used for RT-qPCR experiments, while for all other experiments 4 MMC-AFSC lines and 3 healthy AFSC lines have been used.

Differences between groups were examined for statistical significance using an unpaired Student's *t*-test (when two groups were compared) or ANOVA (when multiple groups were compared). Significance was set at ^∗^*P* < 0.05, ^∗∗^*P* < 0.01, ^∗∗∗^*P* < 0.001, and ^∗∗∗∗^*P* < 0.0001.

## 3. Results

### 3.1. Morphology, Proliferation, and Characterization of MMC-AFSCs

**Figure 1:**
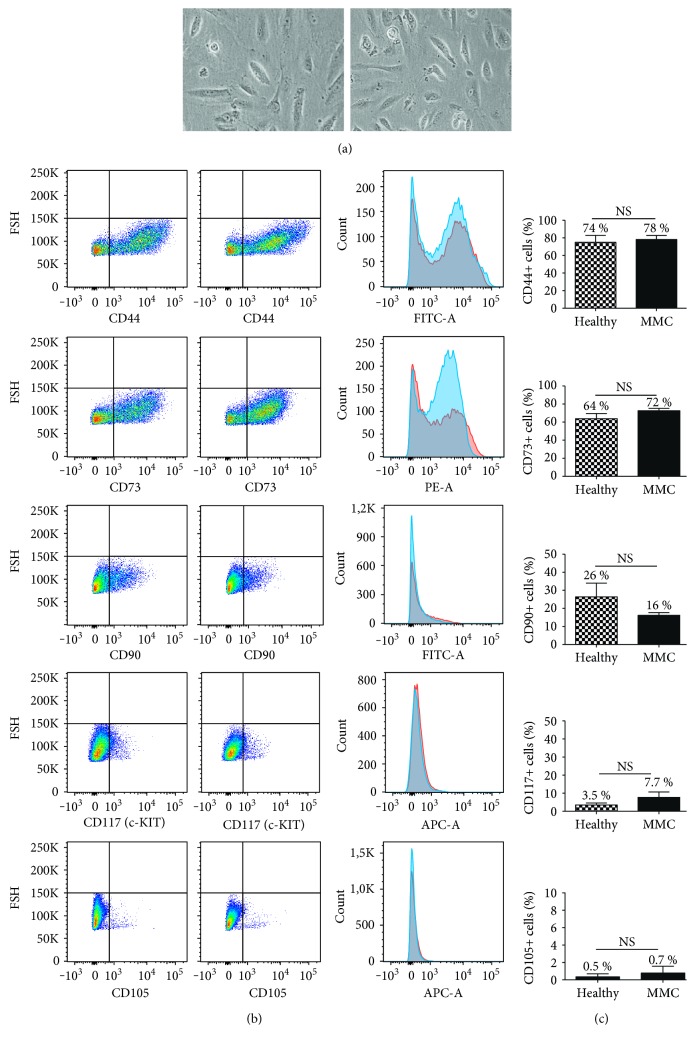
Morphological and fluorescence activated cell-sorting (FACS) analysis of MMC-AFSCs and healthy AFSCs. (a) Microscopy images from MMC-AFSCs and healthy AFSCs before neural induction process. Scale bar = 100 *μ*m. (b) Representative flow cytometry plots and histograms from MMC-AFSCs and healthy AFSCs for the expression of mesenchymal markers CD44, CD73, CD90, CD117, and CD105. (c) Cell surface expression of mesenchymal markers CD44, CD73, CD90, CD117, and CD105 determined in MMC-AFSC and healthy AFSC populations by flow cytometry analysis, shown as percentage. *N* = 4 and values are indicated as mean ± SD. NS, not significant.

We first aimed at assessing the morphological characteristics of AFSCs to address whether differences between MMC-AFSCs and healthy AFSCs were present. No morphological differences could be observed between the two cell types (Figure 1(a)). We then sought whether MMC-AFSCs and healthy AFSCs retained multipotent characteristics. We checked for the expression of previously described mesenchymal stem cell markers [21, 30] on MMC-AFSCs and healthy AFSCs by flow cytometry. Analysis revealed that MMC-AFSCs and healthy AFSCs similarly expressed a set of specific mesenchymal stem cell surface markers, including CD44, CD73, and CD90. Moreover, cells expressed low levels of CD117 (also known as C-Kit) and appeared to be almost negative for CD105 (Figures 1(b) and 1(c)). Therefore, we concluded that MMC-AFSCs and healthy AFSCs had similar morphology and retained multipotent stem cell characteristics.

### 3.2. Characterization of MMC-AFSCs by RT-qPCR

**Figure 2:**
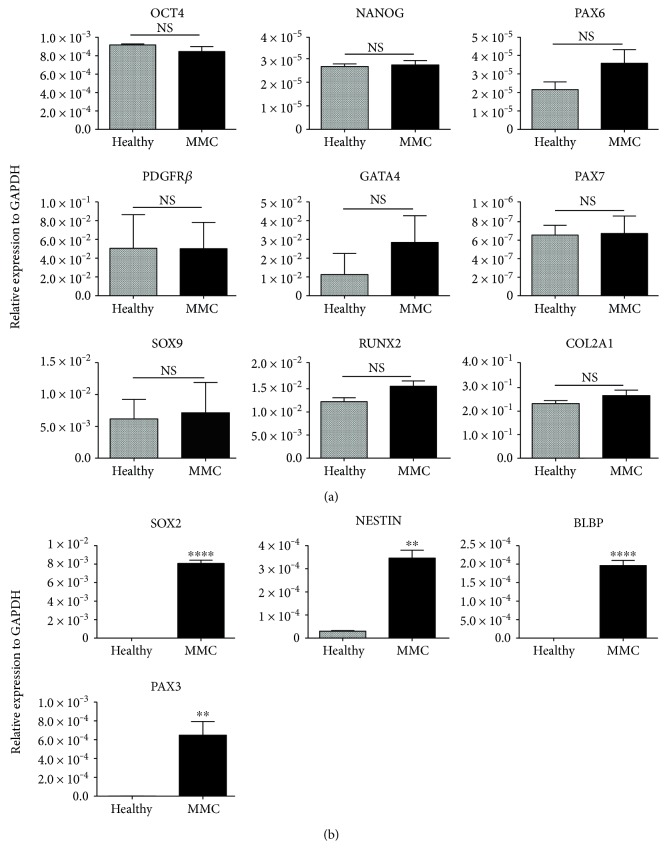
Gene expression analysis of MMC-AFSCs and healthy AFSCs. (a) Expression profile of pluripotency genes *OCT4*, *NANOG*, early neural gene *PAX6,* mesoendodermal genes *PDGFRβ* and *GATA4*, myogenic transcription factor *PAX7*, osteochondrogenic genes *SOX9* and *RUNX2*, and *COL2A1* in MMC-AFSCs and healthy AFSCs by RT-qPCR analysis. Data were represented as relative fold expression, normalized for the housekeeping gene *GAPDH*; NS, not significant. *N* = 4 and values are indicated as mean ± SD. (b) Expression profile of early neural and radial glial genes *SOX2, NESTIN, BLBP*, and *PAX3* in MMC-AFSCs and healthy AFSCs by RT-qPCR analysis. Data were represented as relative fold expression, normalized for the housekeeping gene *GAPDH*; *N* = 4 and values are indicated as mean ± SD. ^∗∗^*P* < 0.01 Healthy versus MMC (NESTIN and PAX3); ^∗∗∗∗^*P* < 0.0001 Healthy versus MMC (SOX2 and BLBP).

We aimed to assess the expression levels of lineage-specific and pluripotent stem cell markers in MMC-AFSCs and healthy AFSCs. The RT-qPCR analysis showed that MMC-AFSCs and healthy AFSCs presented similar low expression profiles of pluripotency genes *OCT4* and *NANOG*, the myogenic transcription factor *PAX7* and the early neural transcription factor *PAX6*. Conversely, similar high expressions of the mesoendodermal genes *PDGFR*𝛽*, GATA4*, osteogenic transcription factor *RUNX2*, and the chondrogenic genes *SOX9* and *COL2A1* was detected in both MMC-AFSCs and healthy AFSCs (Figure 2(a)). Additionally, all AFSC lines presented low expression of mesoendodermal genes *MIXL1, GATA6, SOX17*, and *FOXA1* and negative expression of mesoendodermal and ectodermal genes, including *BRACH, PDGFRa, ACAN, OCN, MYOD, MYH3, RELN, POU4F2*, and *FOXG1* (Supporting information Figure 1(c)). Intriguingly, overexpression of early neural genes *SOX2* and *NESTIN*, the radial glial (RG) gene *BLBP* and the myogenic transcription factor *PAX3* (also known as a neural crest stem cell migration marker) was observed in MMC-AFSCs (Figure 2(b)).

We concluded that MMC-AFSCs presented significantly higher expression levels of neural and RG fate specification genes compared to healthy AFSCs.

### 3.3. Neural Differentiation Potential of MMC-AFSCs

**Figure 3:**
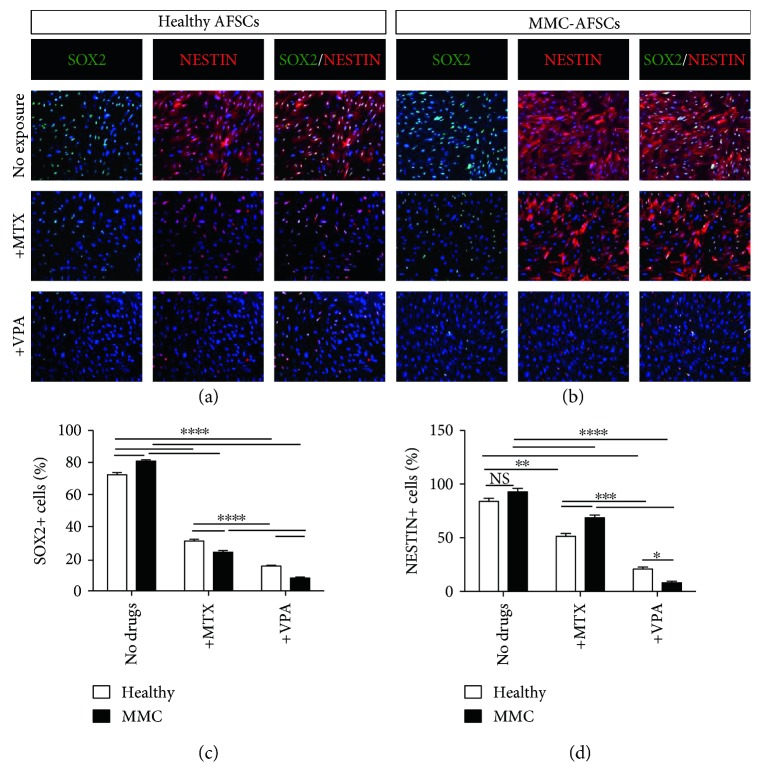
Early neural differentiation potential of MMC-AFSCs and healthy AFSCs. (a, b) IF analysis of MMC-AFSCs and healthy AFSCs at day 15 of neural induction, exposed or not with MTX and VPA, showing positive cells for early neural markers SOX2 (green) and NESTIN (red). Representative images of healthy AFSCs (clone number 1) and MMC-AFSCs (clone number 5) were shown for all healthy and MMC lines. Nuclei were counterstained with Hoechst (blue). *N* = 4, scale bar = 100 *μ*m. (c) Enumeration of SOX2^+^ cells in neural-derived MMC-AFSCs and healthy AFSCs at day 15 of neural induction, exposed or not with MTX and VPA, shown as percentage. *N* = 4 and values are indicated as mean ± SD. ^∗∗∗∗^*P* < 0.0001 Healthy versus Healthy + MTX, Healthy versus Healthy + VPA, Healthy + MTX versus Healthy + VPA, Healthy versus MMC, MMC versus MMC + MTX, MMC versus MMC + VPA, Healthy + MTX versus MMC + MTX, and Healthy + VPA versus MMC+VPA. (d) Enumeration of NESTIN^+^ cells in neural-derived MMC-AFSCs and healthy AFSCs at day 15 of neural induction, exposed or not with MTX and VPA, shown as percentage. *N* = 4 and values are indicated as mean ± SD. NS, not significant. ^∗^*P* < 0.05 Healthy + VPA versus MMC + VPA; ^∗∗^*P* < 0.01 Healthy versus Healthy + MTX; ^∗∗∗^*P* < 0.001 Healthy + MTX versus MMC+MTX, Healthy + MTX versus Healthy + VPA, and MMC + MTX versus MMC + VPA; and ^∗∗∗∗^*P* < 0.0001 Healthy versus Healthy + VPA, MMC versus MMC + MTX, and MMC versus MMC + VPA.

Because of the overexpression of early neural markers in MMC-AFSCs by RT-qPCR, we hypothesized that MMC-AFSCs could have better *in vitro* neural differentiation commitment compared to healthy AFSCs. Therefore, we induced differentiation of MMC-AFSCs and healthy AFSCs towards early neural lineage. Moreover, as the pharmacological agents MTX and VPA are clinically correlated with MMC in fetuses, we sought to explore their *in vitro* effect on early neural-derived MMC-AFSCs and healthy AFSCs. To this end, AFSCs were exposed to either MTX or VPA during the first 3 days of neural induction. At day 15, experiments were stopped and immunofluorescence (IF) analyses were performed in order to evaluate the neural differentiation potential of MMC-AFSCs and healthy AFSCs. At day 15 of neural induction, MMC-AFSCs and healthy AFSCs were double positive for early neural proteins SOX2 and NESTIN (Figures 3(a) and 3(b)), with MMC-AFSCs presenting a significantly higher amount of SOX2^+^ cells. Conversely, upon MTX and VPA exposure, the amount of SOX2^+^ cells was dramatically reduced compared to untreated conditions in both MMC-AFSCs and healthy AFSCs. Interestingly, MMC-AFSCs appeared to be more sensitive to MTX and VPA exposure as evidenced by the significantly reduced amount of SOX2^+^ cells compared to healthy AFSCs (Figure 3(c)). Conversely, MMC-AFSCs and healthy-AFSCs uniformly stained positive for NESTIN (Figures 3(a) and 3(b)). Following quantification, the exposure to either pharmacological agents resulted in a decreased amount of NESTIN^+^ cells, with VPA exposure effect appearing to be more severe in MMC-AFSCs (Figure 3(d)).

**Figure 4:**
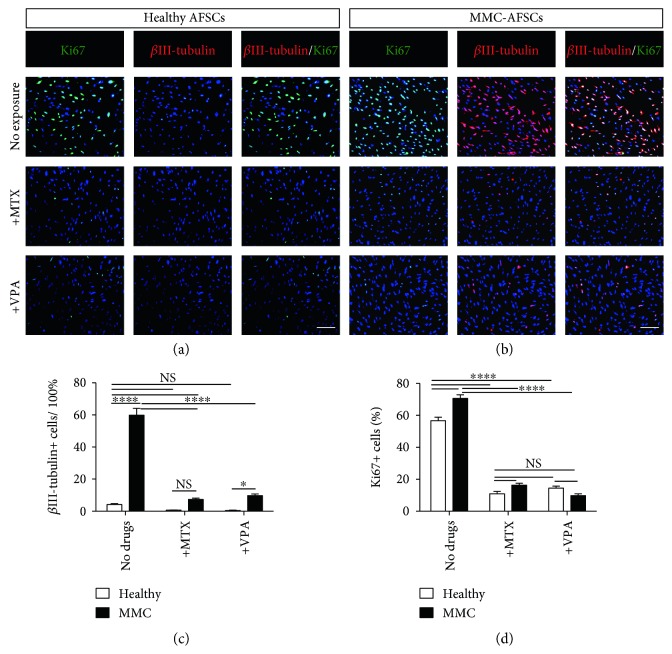
Proliferative and immature neural differentiation potential of MMC-AFSCs and healthy AFSCs. (a, b) IF analysis of MMC-AFSCs and healthy AFSCs at day 15 of neural induction, exposed or not with MTX and VPA, showing positive cells for Ki67 (green) and the immature neural differentiation marker *β*III-tubulin (red). Representative images of healthy AFSCs (clone number 1) and MMC-AFSCs (clone number 5) were shown for all healthy and MMC lines. Nuclei were counterstained with Hoechst (blue). *N* = 4, scale bar = 100 *μ*m. (c) Enumeration of *β*III-tubulin^+^ cells in neural-derived MMC-AFSCs and healthy AFSCs at day 15 of neural induction, exposed or not with MTX and VA, shown as percentage. *N* = 4 and values are indicated as mean ± SD. NS, not significant. ^∗^*P* < 0.05 Healthy + VPA versus MMC + VPA; ^∗∗∗∗^*P* < 0.0001 Healthy versus MMC, MMC versus MMC + MTX, and MMC versus MMC + VPA. (d) Enumeration of Ki67^+^ cells in neural-derived MMC-AFSCs and healthy AFSCs at day 15 of neural induction, exposed or not with MTX and VPA, shown as percentage. *N* = 4 and values are indicated as mean ± SD. NS, not significant. ^∗∗∗∗^*P* < 0.0001 Healthy versus MMC, Healthy versus Healthy + MTX, Healthy versus Healthy + VPA, MMC versus MMC + MTX, and MMC versus MMC + VPA.

We next evaluated the immature neural differentiation potential of MMC-AFSCs and checked the proliferation of neural-derived MMC-AFSCs. To this end, we assessed the subcellular localization of *β*III-tubulin and Ki67. IF analysis demonstrated that double positive *β*III-tubulin and Ki67 cells were much higher in MMC-AFSCs compared to healthy AFSCs (Figures 4(a) and 4(b)). Indeed, approximately 60% of MMC-AFSCs stained positive for *β*II-tubulin, while they were less than 5% in healthy AFSCs. The exposure to MTX and VPA dramatically decreased the number of *β*III-tubulin^+^ cells in MMC-AFSCs (Figure 4(c)). Additionally, analysis revealed that MMC-AFSCs and healthy AFSCs were highly proliferative at day 15 from neural induction (Figures 4(a) and 4(b)). Conversely, a dramatic decrease in the amount of Ki67*^+^* cells was observed in all AFSC lines following exposure to VPA and MTX (Figure 4(d)).

**Figure 5:**
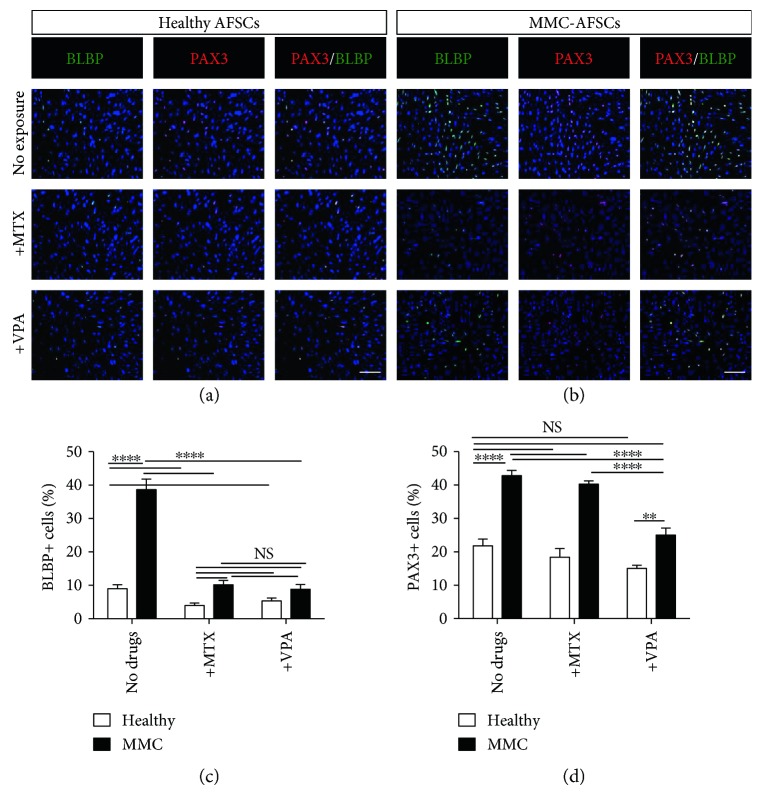
Early radial glial differentiation of MMC-AFSCs and healthy AFSCs. (a, b) IF analysis of MMC-AFSCs and healthy AFSCs and at day 15 of neural induction, exposed or not with MTX and VPA, showing positive cells for the early radial glial marker BLBP (green) and the neural crest stem cell migration marker PAX3 (red). Representative images of healthy AFSCs (clone number 1) and MMC-AFSCS (clone number 5) were shown for all healthy and MMC lines. Nuclei were counterstained with Hoechst (blue). *N* = 4, scale bar = 100 *μ*m. (c) Enumeration of BLBP^+^ cells in neural-derived MMC-AFSCs and healthy AFSCs at day 15 of neural induction, exposed or not with MTX and VPA, shown as percentage. *N* = 4 and values are indicated as mean ± SD. NS, not significant. ^∗∗∗∗^*P* < 0.0001 Healthy versus Healthy + MTX, Healthy versus Healthy + VPA, Healthy versus MMC, MMC versus MMC + MTX, and MMC versus MMC + VPA. (d) Enumeration of PAX3^+^ cells in neural-derived healthy AFSCs and MMC-AFSCs at day 15 of neural induction, exposed or not with MTX and VPA, shown as percentage. *N* = 4 and values are indicated as mean ± SD. NS, not significant. ^∗∗^*P* < 0.01 Healthy + VPA versus MMC + VPA; ^∗∗∗∗^*P* < 0.0001 Healthy versus MMC, MMC versus MMC + VPA, and MMC + MTX versus MMC + VPA.

Further, IF analysis showed that MMC-AFSCs had more BLBP^+^ cells compared to healthy AFSCs (Figures 5(a) and 5(b)). Following quantification of BLBP^+^ cells, it was confirmed that MMC-AFSCs presented significantly higher amount of BLBP^+^ cells compared to healthy AFSCs. Both MTX and VPA severely reduced the amount of BLBP^+^ cells at similar rates (Figure 5(c)).

Finally, IF staining for PAX3 also showed a higher percentage of PAX3^+^ cells present in MMC-AFSCs compared to healthy AFSCs. MTX exposure did not have any effect on MMC-AFSCs and healthy AFSCs as both presented similar amounts of PAX3^+^ cells compared to untreated conditions. Conversely, VPA exposure negatively affected the amount of PAX3^+^ cells only in MMC-AFSCs, with no significant changes in PAX3^+^ cells amount observed in healthy AFSCs (Figures 5(a), 5(b), and 5(d)).

Therefore, MMC-AFSCs exhibited a higher percentage of early neural and RG positive cells at day 15 from neural induction compared to healthy AFSCs. In addition, the exposure to MTX and VPA significantly decreased the number of early neural and RG positive cells in MMC-AFSCs. Intriguingly, PAX3^+^ cells appeared to be resistant to MTX treatment for all cell lines, and VPA affected only PAX3^+^ cell number in MMC-AFSC derivatives.

## 4. Discussion

AFSCs have recently emerged as a novel tool for regenerative medicine purposes. They can be obtained from fetuses with minimal risk and can be used for *in vitro* drug screening purposes [22, 31]. Indeed, the effects of several pharmacological agents on fetal development are also observed in AFSCs used as *in vitro* cell model. In fact, VPA treatment alters proliferation and inhibits neural gene expressions in AFSCs [20]. The use of VPA and MTX during pregnancy interferes with normal neural tube formation by blocking dihydrofolate reductase, methylenetetrahydrofolate reductase, and several other key enzymes of the folate pathway [12]. Thus, the folate pathway is critical for the regulation of the neurulation process, which is also evidenced by the benefit of FA supplementation to avoid abnormal neural tube closure [13].

In this study, we used human AFSCs derived from 7 patients diagnosed with MMC to study the early neural differentiation process and to evaluate the effects of MTX and VPA exposure on neurulation event. Our results revealed that human MMC-AFSCs showed similar growth, proliferative potential, pluripotency gene expression, and mesenchymal stem cell characteristics as healthy AFSCs. Previous studies demonstrated that AFSCs derived from MMC Sprague-Dawley rat model showed significantly increased neural stem cell (NSC) markers, including SOX2 and NESTIN [32, 33]. Moreover, in a recent study, MMC-AFSCs isolated from a rat model were characterized, showing high expression of neural progenitor markers SOX2 and NESTIN [34]. Similarly, in our study, human MMC-AFSCs significantly overexpressed early neural genes *SOX2* and *NESTIN* and in addition *PAX3* when compared to healthy AFSCs. The paired box transcription factor *PAX3* is expressed in neural crest stem cells during vertebral development [35], having a crucial role in neural tube closure as seen in Splotch mutant mouse embryos exhibiting NTDs [36]. In our previous study, we demonstrated the overexpression of *PAX3* gene in a fetal lamb model of MMC [37]. Consistently, our results showed that in humans MMC-AFSCs *PAX3* gene was overexpressed, and the number of PAX3^+^ cells was higher in neural-derived MMC-AFSCs compared to that of the healthy lines. It is possible that upon the failure of neural tube closure, PAX3 progenitors are getting activated in order to force neural crest stem cell migration. Interestingly, MTX exposure did not sort any effect on PAX3 expression in all samples, and VPA exposure slightly affected only the amount of MMC-derived PAX3^+^ cells.

RG cells guide neuronal migration processes and maintain the shape of the cerebral cortex during development. While the increase of the RG marker GFAP has already been described in association with spinal cord injury [38], we sought to analyze the levels of RG marker BLBP in MMC-AFSCs. Intriguingly, BLBP was overexpressed in MMC-AFSCs where, at day 15 from neural induction, the number of BLBP^+^ cells was higher compared to healthy AFSCs. To our knowledge, this is the first report that describes the overexpression of the BLBP gene in human MMC-AFSCs. Intriguingly, our results showed that VPA and MTX exposure negatively affected the amount of SOX2^+^, NESTIN^+^, *β*III-tubulin^+^_,_ and BLBP^+^ cells in AFSC-derived neural progenitors. These data suggested that MTX and VPA severely affected neural and RG commitment. In addition, as expected [39, 40], both drugs negatively impacted cell proliferation, as shown by a decrease on the amount of Ki67^+^ cells.

In summary, MMC-AFSCs retained an enormous potential to differentiate toward early neural progenitors and could be further considered for *in vivo* therapeutic and regenerative applications. Moreover, although samples from more patients would be required to confirm our findings, our results suggested that validation of SOX2, NESTIN, PAX3, and BLBP expression in amniotic fluid could be used as an additional tool to confirm MMC diagnosis.

## Supplementary Material

The information of supplementary materials are as follows: Supporting Information Table S1 Primers List. Supporting information Fig. 1(a,b) Viability assay on AFSCs, exposed to increasing concentrations of MTX and VPA, shown as percentage. N=4 and values are indicated as mean ± SD. ∗∗∗∗P <0.0001 0,1 μM *vs* 0,5 μM and 0,25 μM *vs* 0,5 μM MTX; ∗∗∗∗P <0.0001 0,5 mM *vs* 2 mM and 1 mM *vs* 2 mM VPA. (c) Expression profile by RT-qPCR analysis of meso-endodermal genes: *BRACH*, *PDGFRα*, *MIXL1*, *GATA6*, *SOX17*, *FOXA1*, osteo-chondrogenic genes *ACAN*, *OCN*, myogenic genes *MYH3*, *MYOD* and neural genes *RELN*, *POU4F2* and *FOXG1*. Data were represented as relative fold expression, normalized for the housekeeping gene *GAPDH*. N=3 and values are indicated as mean ± SD. NS, not significant.
