# Supplementary material for: Methotrexate and Valproic Acid Affect Early Neurogenesis of Human Amniotic Fluid Stem Cells from Myelomeningocele
Source: Stem Cells Int. 2017 Sep 13;2017:6101609. doi: 10.1155/2017/6101609 (PMC5615990; doi:10.1155/2017/6101609)
Supplement: Supplementary file 1 — The information of supplementary materials are as follows: Supporting Information Table S1 Primers List. Supporting information Fig. 1(a,b) Viability assay on AFSCs, exposed to increasing concentrations of MTX and VPA, shown as percentage. N=4 and values are indicated as mean ± SD. ∗∗∗∗P <0.0001 0,1 μM vs 0,5 μM and 0,25 μM vs 0,5 μM MTX; ∗∗∗∗P <0.0001 0,5 mM vs 2 mM and 1 mM vs 2 mM VPA. (c) Expression profile by RT-qPCR analysis of meso-endodermal genes: BRACH, PDGFRα, MIXL1, GATA6, SOX17, FOXA1, osteo-chondrogenic genes ACAN, OCN, myogenic genes MYH3, MYOD and neural genes RELN, POU4F2 and FOXG1. Data were represented as relative fold expression, normalized for the housekeeping gene GAPDH. N=3 and values are indicated as mean ± SD. NS, not significant. [file 6101609.f1.pdf]

Supporting Information Table S1
Primers List

Gene	Forward primer	Reverse primer
GAPDH	TCAAGAAGGTGGTGAAGCAGG	ACCAGGAAATGAGCTTGACAAA
OCT4	CGAGCAATTTGCCAAGCTCCTGAA	GCCGCAGCTTACACATGTTCTTGA
SOX2	TGGCGAACCATCTCTGTGGT	CCAACGGTGTCAACCTGCAT
NANOG	TGGCCGAAGAATAGCAATGGTGTG	TTCCAGGTCTGGTTGCTCCACATT
PAX6	GTGTCCAACGGATGTGTGAG	CTAGCCAGGTTGCGAAGAAC
BLBP	GGACTCTCAGCACATTCAAGAA	CCACATCACCAAAAGTAAGGGT
NESTIN	CAGCGTTGGAACAGAGGTTGG	TGGCACAGGTGTCTCAAGGGTAG
PAX3	GCCGCATCCTGAGAAGTAAA	CTTCATCTGATTGGGGTGC
PAX7	GAGGATGAAGCGGACAAGAA	TCAGTGGGAGGTCAGGTT
COL2A1	GGCAATAGCAGGTTCACGTACA	CGATAACAGTCTTGCCCCACTT
RUNX2	CATGGTGGAGATCATCGC	ACTCTTGCCTCGTCCACTC
POU4F2	GGGCAAGAGCCATCCTTTCAA	CTGTTCATCGTGTGGTACGTG
RELN	GTAGCAAGCCCTTCAGCAAC	CCCTGAGGCCAGTACAACAT
PDGFRA	GATTAAGCCGGTCCCAACCT	GGATCTGGCCGTGGGTTT
MYH3	GCCCTTTGACATTCGCACTG	CGGGACAAAATCTTGGCTTTGA
PDGFRB	TGGCAGAAGAAGCCACGTT	GGCCGTCAGAGCTCACAGA
OCN	ATGAGAGCCCTCACACTCCTC	GCCGTAGAAGCGCCGATAGGC
SOX9	GACTTCCGCGACGTGGAC	GTTGGGCGGCAGGTACTG
ACAN	TCGAGGACAGCGAGGCC	TCGAGGGTGTAGCGTGTAGAGA
MIXL1	GGATCCAGGTATGGTTCCAG	CATGAGTCCAGCTTTGAACC
GATA4	TCCAAACCAGAAAACGGAAG	CTGTGCCCGTAGTGAGATGA
FOXA1	AGGCCTACTCCTCCGTCCCG	CTAGGCCCGGGTTGGCATAGG
DLX5	CGCCTCGCTGGGATTG	CTTGATCTTGGATCTTTTGTTCTGAA
SOX17	CGCTTTCATGGTGTGGGCTAAGGACG	TAGTTGGGGTGGTCCTGCATGTGCTG
GATA6	CTCAGTTCCTACGCTTCGCAT	GTCGAGGTCAGTGAACAGCA
FOXA1	GCAATACTCGCCTTACGGCT	TACACACCTTGGTAGTACGCC
BRACH	ACCCAGTTCATAGCGGTGAC	AAGCTTTTGCAAATGGATTG
MYOD	CGCCATCCGCTATATCGAGG	CTGTAGTCCATCATGCCGTCG
FOXG1	GAGCGACGACGTGTTCATC	GCCGTTGTAACTCAAAGTGCTG
