# Supplementary figures and images for: Methotrexate and Valproic Acid Affect Early Neurogenesis of Human Amniotic Fluid Stem Cells from Myelomeningocele
Source: Stem Cells Int. 2017 Sep 13;2017:6101609. doi: 10.1155/2017/6101609 (PMC5615990; doi:10.1155/2017/6101609)

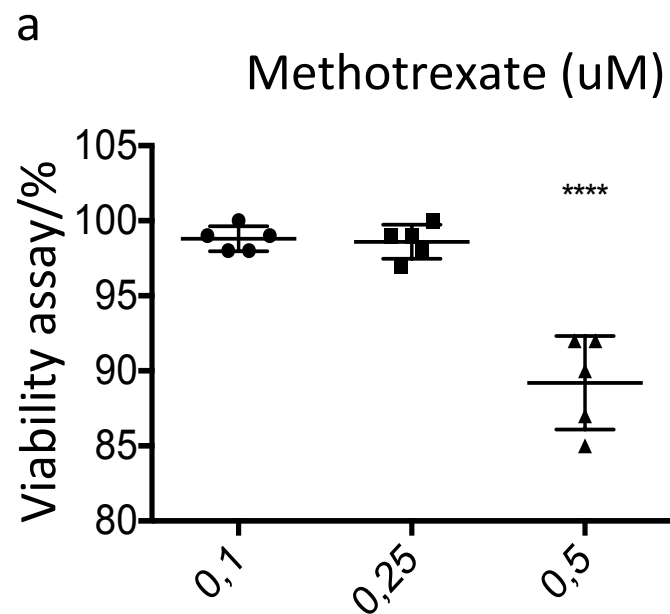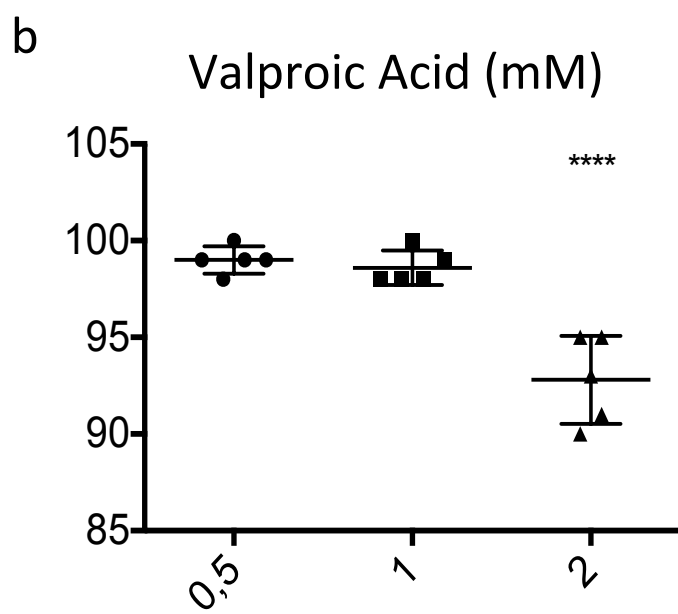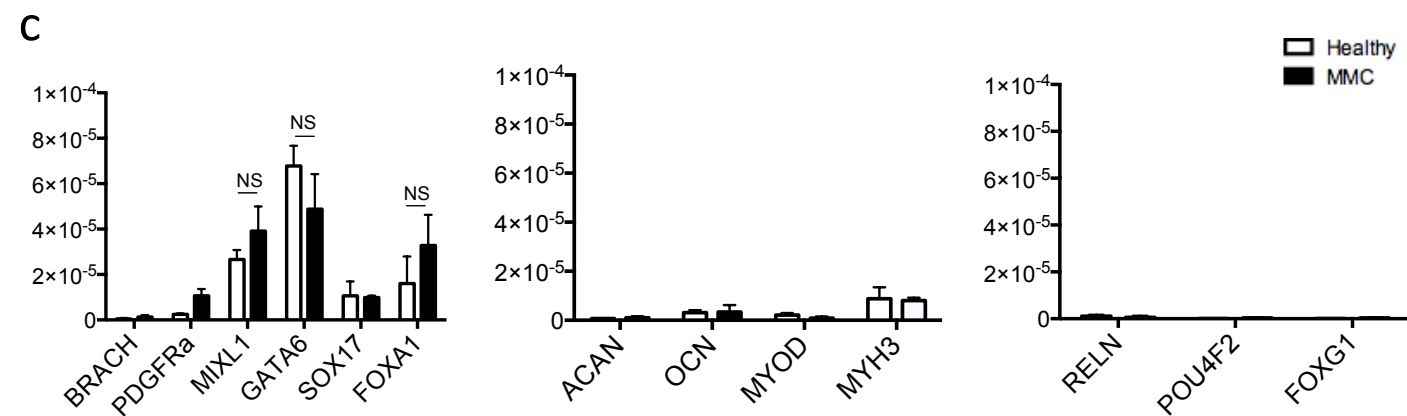

Supplement: Supplementary file 2 [file 6101609.f2.pdf]
